# Role of the TME in immune checkpoint blockade resistance of non-small cell lung cancer

**DOI:** 10.20517/cdr.2024.166

**Published:** 2024-12-16

**Authors:** Yuening Dai, Xueqi Tian, Xuanting Ye, Yabin Gong, Ling Xu, Lijing Jiao

**Affiliations:** ^1^Department of Oncology I, Yueyang Hospital of Integrated Traditional Chinese and Western Medicine, Shanghai University of Traditional Chinese Medicine, Shanghai 200437, China.; ^2^Institute of Translational Cancer Research for Integrated Chinese and Western Medicine, Yueyang Hospital of Integrated Traditional Chinese and Western Medicine, Shanghai University of Traditional Chinese Medicine, Shanghai 200437, China.

**Keywords:** Lung cancer, tumor microenvironment, immune checkpoint blockade, resistance, mechanism

## Abstract

Primary and secondary resistance to immune checkpoint blockade (ICB) reduces its efficacy. The mechanisms underlying immunotherapy resistance are highly complex. In non-small cell lung cancer (NSCLC), these mechanisms are primarily associated with the loss of programmed cell death-ligand 1 (PD-L1) expression, genetic mutations, circular RNA axis and transcription factor regulation, antigen presentation disorders, and dysregulation of signaling pathways. Additionally, alterations in the tumor microenvironment (TME) play a pivotal role in driving immunotherapy resistance. Primary resistance is mainly attributed to TME alterations, including mutations and co-mutations, modulation of T cell infiltration, enrichment of M2 tumor-associated macrophages (M2-TAMs) and mucosal-associated invariant T (MAIT) cells, vascular endothelial growth factor (VEGF), and pulmonary fibrosis. Acquired resistance mainly stems from changes in cellular infiltration patterns leading to “cold” or “hot” tumors, altered interferon (IFN) signaling pathway expression, involvement of extracellular vesicles (EVs), and oxidative stress responses, as well as post-treatment gene mutations and circadian rhythm disruption (CRD). This review presents an overview of various mechanisms underlying resistance to ICB, elucidates the alterations in the TME during primary, adaptive, and acquired resistance, and discusses existing strategies for overcoming ICB resistance.

## INTRODUCTION

At the global level, lung cancer remains to be the main cause of cancer-related deaths. Lung cancer is the second greatest cause of cancer death, taking the lives of almost 350 people every day. Furthermore, lung cancer ranks as the foremost cause of both male and female cancer fatalities in the United States^[1]^. Currently, immune checkpoint inhibitors (ICIs) have been extensively employed in perioperative or non-surgical patients with non-small cell lung cancer (NSCLC). Whether combined with chemotherapy or not, ICIs have emerged as a treatment standard. Immunotherapy has made significant advancements in recent years, particularly through ICIs, including widely utilized agents such as cytotoxic T-lymphocyte-associated protein-4 (CTLA-4), programmed death-1 (PD-1), and programmed cell death-ligand 1 (PD-L1). Other forms of immunotherapy include therapeutic vaccines, immunomodulators, autologous cell therapy, *etc.*^[2,3]^. However, immunotherapy used in clinical settings frequently results in poor response rates and unavoidable primary or secondary drug resistance.

This review provides a comprehensive overview of the tumor microenvironment (TME)-related mechanisms underlying immune checkpoint blockade (ICB) resistance in NSCLC. Primary resistance is mainly attributed to TME alterations, which involve mutations and co-mutations, modulation of T cell infiltration, enrichment of M2 tumor-associated macrophages (M2-TAMs), vascular endothelial growth factor (VEGF), pulmonary fibrosis and mucosal-associated invariant T (MAIT) cells. Acquired resistance mainly stems from changes in cellular infiltration patterns leading to “cold” or “hot” tumors, altered interferon (IFN) signaling pathway expression, involvement of extracellular vesicles (EVs), and oxidative stress responses, as well as post-treatment gene mutations and circadian rhythm disruption (CRD).

## THERAPEUTIC RESISTANCE TO ICIS IN NSCLC

ICIs have been extensively utilized in clinical therapy. ICIs reinvigorate T cell activation by obstructing the inhibitory interactions, such as PD-1 and PD-L1^[4]^. IMpower-010^[5]^ demonstrated that among postoperative NSCLC patients with stage II-IIIA, atezolizumab significantly enhanced disease-free survival (DFS) compared to the best supportive treatment after adjuvant chemotherapy(42.3 mo *vs.* 35.3 mo). It also exhibited a significant benefit in the subgroup of tumor cells expressing PD-L1 ≥ 50%, while the benefit was less pronounced in tumor cells expressing PD-L1 in 1%-49%. The KEYNOTE-091 (PEARLS) trial^[6]^, similar to the aforementioned study design, revealed that NSCLC patients with stage IB (≥ 4 cm) to stage IIIA who underwent surgical resection and received adjuvant chemotherapy along with up to one year of adjuvant pembrolizumab therapy experienced improved DFS (53.6 mo *vs.* 42 mo). The CheckMate 816^[7]^ study demonstrated that the addition of neoadjuvant nivolumab to chemotherapy significantly extended event-free survival (EFS) (31.6 mo *vs.* 20.8 mo) and enhanced major pathologic response (MPR) rates (24% *vs.* 2.2%). After chemoradiotherapy, durvalumab significantly extended progression-free survival (PFS) and overall survival (OS) in stage III NSCLC patients as compared to placebo^[8,9]^. Hence, durvalumab is presently authorized for the treatment of inoperable stage III NSCLC following chemoradiotherapy, even in cases where tumor PD-L1 expression is minimal.

Meanwhile, dual ICB combinations have emerged as a highly promising treatment strategy in recent years. Checkmate 227^[10]^ indicated that in patients with PD-L1 ≥ 1%, the utilization of dual ICB was associated with a prolonged PFS (17.1 mo *vs.* 14.9 mo) compared to chemotherapy. Subsequently, Checkmate 9LA^[11]^ demonstrated that dual ICB in combination with two-cycle chemotherapy conferred benefits to patients irrespective of PD-L1 expression level, with a significantly longer median OS (15.6 mo *vs.* 10.9 mo) in contrast to chemotherapy alone. Recently, substantial progress has also been achieved in dual immunotherapy combination trials. The COAST trial^[12]^ revealed that the use of anti-PD-L1 in combination with either oleclumab (anti-CD73) or monalizumab (anti-NGK2A) following concurrent radiotherapy and chemotherapy in unresectable stage III NSCLC led to objective response rate (ORR) and PFS benefits, which holds great significance for conducting further studies with larger sample sizes. A phase II clinical trial with a sample size of 309 patients^[13]^ discovered that the combination of nivolumab with relatlimab and cisplatin-based doublet chemotherapy was superior to nivolumab in combination with cisplatin-based chemotherapy, with more pronounced benefits for patients with PD-L1 ≥ 1% and non-squamous cell carcinoma.

Immunotherapies are in the spotlight due to a series of Food and Drug Administration (FDA) approvals. Although ICIs have shown good improvement in OS of patients with NSCLC, the response rate is about 20%-30%^[14,15]^. A multicenter retrospective study involving 1,127 patients found that secondary resistance occurred in patients who had tumor control after an average of 26 months of ICI treatment and stopped taking ICIs, with a median time of 10 months for progression after cessation^[16]^. It is thus unavoidable that both oncologists and patients will have to confront the issue of primary and secondary resistance to immunotherapy.

Tumor drug resistance is not solely a result of the tumor’s confrontation with the immune system but rather arises from the intricate interplay among the host, tumor cells, and immune microenvironment. The patient’s immune response undergoes dynamic and continuous changes due to various therapeutic interventions such as surgery, chemotherapy, radiotherapy, and immunotherapy^[17]^. Consequently, the mechanism of drug resistance also exists in a state of constant flux. Numerous complex factors, including alterations in the microenvironment and the emergence of gene mutations in tumor cells under treatment pressure, may contribute to the development of drug resistance. The Society for Immunotherapy of Cancer (SITC) previously defined single PD-1 resistance based on minimal drug exposure requirements, best response, primary resistance, secondary resistance, and disease progression after treatment discontinuation^[18]^. According to the recommendations of the 1st meeting held by SITC immunotherapy resistance, primary resistant patients are defined as those whose tumors are assessed as progressive disease (PD) or stable disease (SD) within less than 6 months after receiving immunotherapy for a minimum duration of 6 weeks. On the other hand, secondary resistance is characterized by tumors that initially respond to immunotherapy and achieve complete response (CR), partial remission (PR), or SD for more than 6 months but eventually progress according to imaging scans^[18]^. In 2021, SITC also established the definition of ICI-chemotherapy combined resistance, which considered primary resistant patients as those evaluated with PD within 6 months after treatment lasting at least 6-8 weeks. Secondary resistance is identified when PD occurs following treatment for a minimum duration of 6 months^[19]^.

Clinically, drug resistance mechanisms can be mainly categorized into two types: those that hinder patients’ response to immunotherapy and those that facilitate relapse following initial treatment success. Tumor cells employ similar or overlapping mechanisms to evade antitumor immune responses^[17]^. The mechanism underlying immunotherapy resistance is highly intricate and not yet fully elucidated. Several fundamental mechanisms that contribute to tumor resistance have been identified, such as activation of the mitogen-activated protein kinase (MAPK) signaling pathway and impaired T cell response caused by diminished tumor antigen expression^[17]^. Experimental results demonstrate the existence of immune surveillance in the human body, wherein the process of tumor eradication primarily involves four steps. Upon receiving an alarm from immune cells, endogenous IFN-γ facilitates the rejection response between the immune system and tumors. Tumor cells serve as crucial targets for IFN-γ during this rejection reaction. By upregulating components of the major histocompatibility complex I (MHC I) antigen processing and presentation pathway, IFN-γ enhances the immunogenicity of tumor cells. Additionally, at the tumor site, IFN-γ induces local production of chemokines that recruit more innate immune system cells to target tumors. Simultaneously, a positive feedback loop involving natural killer (NK) cells stimulates increased production of IFN-γ to activate processes such as anti-proliferation, pro-apoptosis, and vascular inhibition for effective tumor elimination. Activation of dendritic cells (DCs) triggers CD4^+^ and CD8^+^ responses that directly participate in killing tumor cells within the body^[20]^.

However, the incidence of primary resistance is higher in trials using immune monotherapy as first-line treatment. The incidence rate for primary resistance to second-line immune monotherapy or combined immunotherapy ranges from 26.9% to 44%, while it is between 3% and 27% for first-line monotherapy or combined immunotherapy. Notably, the primary resistance incidence rate is lower with combined immunotherapy than with immune monotherapy [Table 1].

**Table 1:** Landmark trials in immunotherapy for the treatment of NSCLC

**Author/year/ trial number**	**Trial name**	**Treatment regimen**	***N***	**Phase**	**Pathology**	**Clinical stage and treatment line**	**PD-L1 expression**	**Outcomes**			**Primary resistance (%)^D^**
								**ORR (%)**	**mPFS (mo)**	**mOS (mo)**	
**Adjuvant therapy**											
O’Brien *et al.*^[6]^ 2022 (NCT02504372)	Keynote 091	Pembro *vs.* placebo	1,177	III	NSQ (*n* = 761) & SQ (*n* = 416)	IB-IIIA; consolidation	All	UK	53.6 *vs.* 42.0^*^^,^^B^	NR	UK
Felip *et al.*^[5]^ 2021 (NCT02486718)	IMpower010	Atezo (PD-L1) *vs.* best supportive care	1,005	III	NSQ (*n* = 659) & SQ (*n* = 346)	IB-IIIA; 1st	All	UK	UK	NR (5-year: 74.8%)	UK
**Advanced first-line**											
Carbone *et al.*^[21]^ 2017 (NCT02041533)	Checkmate 026	Nivo *vs.* chemo	423	III	NSQ (*n* = 412) & SQ (*n* = 129)	IV; 1st	PD-L1 ≥ 1%	26 *vs.* 33^A^	4.2 *vs.* 5.8	13.7 *vs.* 13.8	27
							PD-L1 ≥ 5%	UK	4.2 *vs.* 5.9^A^	14.4 *vs.* 13.2	
Hellmann *et al.*^[10]^ 2019 (NCT02477826)	Checkmate 227	Nivo + ipilim *vs*. nivo *vs.* chemo	1,189	III	NSQ (*n* = 839) & SQ (*n* = 350)	IV; 1st	PD-L1 ≥ 1%	35.9 *vs.* 27.5 *vs.* 30^*^	17.1 *vs.* 15.7 *vs.* 14.9^*^	5.1 *vs.* 4.2 *vs.* 5.6	22.7
Paz-Ares *et al.*^[11]^ 2021 (NCT03215706)	Checkmate9LA	Nivo + ipilim + chemo *vs.* chemo	719	III	NSQ (*n* = 495) & SQ (*n* = 224)	IV; 1st	All	37.7 *vs.* 25.1	6.8 *vs.* 5.0^*^	15.6 *vs.* 10.9^*^	9
Reck *et al.*^[22,23]^ 2016 (NCT02142738)	Keynote 024	Pembro *vs.* chemo	305	III	NSQ (*n* = 249) & SQ (*n* = 56); EGFR/ALK excluded	IV; 1st	PD-L1 ≥ 50%	44.8 *vs.* 27.8	10.3 *vs.* 6.0^*^	26.3 *vs.* 13.4	22.70
Mok *et al.*^[24,25]^ 2019 (NCT02220894)	Keynote 042	Pembro *vs.* chemo	1,275	III	NSQ (*n* = 782) & SQ (*n* = 492); EGFR/ALK excluded	Advanced; 1st	PD-L1 ≥ 1%	27 *vs.* 27	5.4 *vs.* 6.5	16.7 *vs.* 12.1^*^	20.90
							PD-L1 ≥ 20%	33 *vs.* 29	6.2 *vs.* 6.6	17.7 *vs.* 13^*^	
							PD-L1 ≥ 50%	39 *vs.* 32	7.1 *vs.* 6.4^*^	20 *vs.* 12.2^*^	
Langer *et al.*^[26,27]^ 2016 (NCT02039674)	Keynote 021	Pembro + chemo *vs.* chemo	123	II	NSQ; EGFR/ALK excluded	IIIB or IV; 1st	All	55 *vs.* 29	13 *vs.* 8.9^*^	34.5 *vs.* 21.1	3
Gandhi *et al.*^[28]^ 2018 (NCT02578680)	Keynote 189	Pembro + chemo *vs.* chemo	616	III	NSQ	Advanced; 1st	All	47.6 *vs.* 18.9	8.8 *vs.* 4.9^*^	-*vs.* 11.3^*^	9
							PD-L1 < 1%	32.3 *vs.* 14.3	6.1 *vs.* 5.1	61.7% *vs.* 52.2%^C^	
							PD-L1 1%-49%	48.4 *vs.* 20.7	UK	71.5% *vs.* 50.9%^C^	
							PD-L1 ≥ 50%	61.4 *vs.* 22.9	UK	73% *vs.* 48.1%^C^	
Paz-Ares *et al.*^[29]^ 2018 (NCT02775435)	Keynote 407	Pembro + C + P *vs.* C + P	559	III	SQ	IV; 1st	All	57.9 *vs.* 38.4	6.4 *vs.* 4.8^*^	15.9 *vs.* 11.3^*^	6.10
							PD-L1 < 1%	63.2 *vs.* 40.4	6.3 *vs.* 5.3	15.9 *vs.* 10.2	
							PD-L1 1%-49%	49.5 *vs.* 41.3	7.2 *vs.* 5.2	14 *vs.* 11.6	
							PD-L1 ≥ 50%	60.3 *vs.* 32.9	8 *vs.* 4.2	NR *vs.* NR	
Boyer *et al.*^[30]^ 2020 (NCT03302234)	Keynote 598	Pembro + ipilim *vs.* placebo	568	III	NSQ (*n* = 410) & SQ (*n* = 158)	IV; 1st	PD-L1 ≥ 50%	45.4 *vs.* 45.4	8.2 *vs.* 8.4	21.4 *vs.* 21.9	18
Lu *et al.*^[31]^ 2020 (NCT03663205)	RATIONALE 304	Tisle + AC/P *vs*. AC/P	334	III	NSQ	IIIB or IV; 1st	All	57 *vs.* 36.9	9.7 *vs.* 7.6^*^	UK	UK
							PD-L1 < 1%	41.7 *vs.* 27.1	7.6 *vs.* 7.6		
							PD-L1 1%-49%	64.2 *vs.* 48.1	9.8 *vs.* 8		
							PD-L1 ≥ 50%	73 *vs.* 41.7	11.5 *vs.* 4.6		
Zhou *et al.*^[32]^ 2020 (NCT03629925)	ORIENT-12	Sinti + chemo *vs.* placebo + chemo	357	III	SQ	IIIB/IIIC or IV; 1st	All	44.7 *vs.* 35.4	5.1 *vs.* 4.9^*^	NR	10.10
Herbst *et al.*^[12]^ 2022 (NCT03822351)	COAST	Durva + oleclu *vs.* durva + mona *vs.* durva	189	II	NSQ (*n* = 108) & SQ (*n* = 81)	III; 1st	All	30 *vs.* 35.5 *vs.* 17.9	NR *vs.* 15.1 *vs.* 6.3	UK	10/6.5/16.4
Girard *et al.*^[13]^ 2024 (NCT04623775)	RELATIVITY-104	Nivo + rela + chemo *vs.* nivo + chemo	309	II	NSQ (*n* = 211) & SQ (*n* = 98)	IV; 1st	All	51.3 *vs.* 43.7	UK	UK	UK
							PD-L1 < 1%	50 *vs.* 44.8	5.6 *vs.* 5.8	UK	UK
							PD-L1 ≥ 1%	53.2 *vs.* 40.8	9.8 *vs.* 6.1	UK	UK
**Advanced second-line and beyond**											
Wu *et al.*^[33]^ 2019 (NCT02613507)	Checkmate 078	Nivo *vs.* doce	504	III	NSQ (*n* = 304) & SQ (*n* = 200); EGFR/ALK excluded	IIIB or IV; 2nd	All	17 *vs.* 4	2.8 *vs.* 2.8^*^	12.0 *vs.* 9.6^*^	37.90
							PD-L1 < 1%	UK	UK	11.4 *vs.* 10.2	
							PD-L1 ≥ 1%	UK	UK	12.3 *vs.* 7.9	
Brahmer *et al*.^[34]^ 2015 (NCT01642004)	Checkmate 017	Nivo *vs.* doce	272	III	SQ	IIIB or IV; 2nd	All	20 *vs.* 9	3.5 *vs.* 2.8^*^	9.2 *vs.* 6.0^*^	41
							PD-L1 < 5%	15 *vs.* 12	2.2 *vs.* 2.9	8.5 *vs.* 6.1	
							PD-L1 ≥ 5%	21 *vs.* 8	4.8 *vs.* 3.1	10 *vs.* 6.4	
Borghaei *et al.*^[35]^ 2015 (NCT01673867)	Checkmate 057	Nivo *vs.* doce	582	III	NSQ	IIIB or IV; 2nd	All	19 *vs.* 12	2.3 *vs.* 4.2	12.2 *vs.* 9.4^*^	44
							PD-L1 < 5%	10 *vs.* 14	2.1 *vs.* 4.2^*^	9.8 *vs.* 10.1^*^	
							PD-L1 ≥ 5%	36 *vs.* 13	5.0 *vs.* 3.8^*^	19.4 *vs.* 8.4^*^	
Zhou *et al*.^[36]^ 2021 (NCT03663205)	RATIONALE 303	Tisle *vs.* doce	805	III	NSQ (*n* = 435) & SQ (*n* = 370)	Advanced; ≥ 2nd	All	21.9 *vs.* 7	4.1 *vs.* 2.6^*^	17.2 *vs.* 11.9^*^	37
							PD-L1 ≥ 25%	37.4 *vs.* 6.9	UK	19.1 *vs.* 11.9	
							PD-L1 < 25%	UK	UK	15.2 *vs.* 12.3	
Shi *et al.*^[37]^ 2017 (NCT03150875)	ORIENT-3	Sinti *vs.* doce	290	III	SQ	IIIB/IIIC or IV; ≥ 2nd	All	25.5 *vs.* 2.2	4.3 *vs.* 2.79^*^	11.79 *vs.* 8.25^*^	26.90
Rittmeyer *et al.*^[38]^ 2019 (NCT02008227)	OAK	Atezo *vs.* doce	850	III	NSQ (*n* = 628) & SQ (*n* = 222)	III or IV; ≥ 2nd	All	14 *vs.* 13	2.8 *vs.* 3.0	13.8 *vs.* 9.6	44
**Advanced consolidation**											
Garon *et al.*^[39]^ 2015 (NCT01295827)	Keynote 001	Pembro	495	I	NSQ (*n* = 401) & SQ (*n* = 85) & ANSQ (*n* = 7)	Advanced; consolidation	All	19.40	3.7	12	34.50
							PD-L1 < 1%	10.00	2.1	6.7	
							PD-L1 1%-49%	9.30	2.1	5.9	
							PD-L1 ≥ 50%	34.20	4.5	13.7	
Herbst *et al.*^[40]^ 2016 (NCT01905657)	Keynote 010	Pembro (L) *vs.* pembro (H) *vs.* doce	1,034	II/III	NSQ (*n* = 724) & SQ (*n* = 222) & other (*n* = 25) & UK (*n* = 10)	Advanced; consolidation	PD-L1 ≥ 1%	30 *vs.* 29 *vs.* 8	3.9 *vs.* 4.0 *vs.* 4.0	14.9 *vs.* 17.3 *vs.* 8.2	UK
Antonia *et al.*^[9]^ 2017 (NCT02125461)	PACIFIC	Durva (PD-L1) *vs.* placebo	709	III	NSQ (*n* = 387) & SQ (*n* = 326)	III; consolidation	All	28.4 *vs.* 16.0	16.8 *vs.* 5.6	447.5 *vs.* 29.1	16.50

^*^P < 0.05. ^A^PFS and OS among patients with a programmed death ligand 1 expression level of 5% or more; ^B^DFS as the primary outcome measure; ^C^12-month OS rate; ^D^Primary resistance defined as the patient exhibited no discernible response to immunotherapy, and the tumor failed to exhibit any positive response to immunotherapy^[17]^. NSCLC: Non-small cell lung cancer; PD-L1: programmed cell death-ligand 1; ORR: objective response rate; mPFS: median progress-free survival; mOS: median overall survival; NSQ: non-squamous; SQ: squamous; UK: unknown; NR: not-reached; EGFR: epidermal growth factor receptor; ALK: anaplastic lymphoma kinase; ANSQ: adenosquamous; PFS: progression-free survival; OS: overall survival; DFS: disease-free survival.

## MECHANISMS OF IMMUNOTHERAPY RESISTANCE IN NSCLC

### Loss of PD-L1 expression

PD-L1 expression and tumor mutational burden (TMB) have been demonstrated as valuable biomarkers for the selection of ICB in lung cancer^[41]^. High TMB can enhance the efficacy of immunotherapy in terms of OS for NSCLC patients^[42]^. CheckMate 026^[21]^ and CheckMate 227^[10]^ studies have shown that high TMB is related to significantly improved PFS. PD-L1 expression has an impact on the effectiveness of PD-L1 antibody treatment in tumors with high TMB^[41]^. However, when utilizing anti-CTLA4 or anti-PD-1/CTLA-4 combination therapy, the response may not rely on PD-L1 expression. Currently, there is an absence of a well-established disease-specific TMB threshold for effectively predicting the response of various malignant tumors.

### Genetic mutations

#### Epidermal growth factor receptor

Epidermal growth factor receptor (EGFR), a transmembrane protein with intracellular kinase activity, is expressed in more than 60% of NSCLC cases^[43]^. Single-point mutations can induce EGFR to shift from an inactive state to an active one, altering its signaling pathway and subsequently leading to cell proliferation, angiogenesis, and cancer cell invasion^[44]^. Distinct from EGFR mutation-targeted therapy, the biomarker for immunotherapy undergoes dynamic changes. The poor clinical efficacy of ICIs in EGFR-mutated NSCLC is closely associated with specific TME, TMB, and PD-L1 expression levels^[45]^. A meta-analysis^[46]^ evaluated the efficacy of ICIs utilized in advanced-stage, second-line EGFR-mutated NSCLC, encompassing Checkmate 057, Keynote 010, and POPLAR trials, demonstrating that ICIs, when compared with chemotherapy, did not confer significant OS benefits for patients with EGFR mutations (*P* = 0.42). Meanwhile, another meta-analysis^[47]^ encompassed the OAK and Checkmate 17 trials and discovered that ICIs were correlated with OS prolongation in the EGFR wild-type (EGFR WT) subpopulation (*P* < 0.001) and the Kirsten rat sarcoma viral oncogene homolog (KRAS) mutant subpopulation (*P* = 0.03), yet no significant disparity was identified in the EGFR mutant subpopulation (*P* = 0.54) or the KRAS WT subpopulation (*P* = 0.24). Additionally, the efficacy of immunotherapy is intimately related to the subtype of EGFR mutation, and rare EGFR mutations might be associated with a poorer prognosis^[48]^. Hastings *et al.* retrospectively analyzed the clinical data of 171 EGFR mutation patients who had utilized ICIs and the sequencing data of 383 case cohorts and found that the overall response rate and OS of EGFR exon19 deletion tumors were conspicuously lower than those of EGFR WT tumors (76.7% *vs.* 47%), and the PFS of EGFR exon19 deletion tumors and EGFR L858R mutant tumors was significantly inferior (*P* < 0.001)^[49]^.

In addition, TMB was notably reduced in EGFR exon19 deletion tumors compared to EGFR L858R mutant tumors. Analogous to this finding, Zhou *et al.* discovered that PD-1 expression and CD8^+^PD-1^+^ exhausted T cell infiltration were significantly augmented in patients with EGFR L858R mutation in contrast to patients with EGFR exon19 mutation, along with a superior outcome of PD-1/PD-L1 blockade, which might be related to a higher TMB. Moreover, it was found that lymphocyte activation gene-3 (LAG-3) was conspicuously upregulated following tyrosine kinase inhibitor (TKI) treatment. This furnishes some theoretical basis for the application of LAG-3 inhibitors^[50]^. Meanwhile, EGFR-mutated cells restrain T cell proliferation and related functions and exhibit significantly enhanced resistance to T cell killing. Thus, based on the results of an experiment, neither anti-PD-L1 nor anti-CD73 drugs alone could suppress tumor growth. However, compared with EGFR WT tumors, EGFR-mutated NSCLC expresses higher levels of CD73. The combined use of the two antibodies on EGFR-mutated NSCLC increases the number of tumor-infiltrating CD8^+^ T cells and enhances the production of IFN-γ and tumor necrosis factor-α (TNF-α), which can prominently inhibit tumor growth^[51]^, providing a theoretical foundation for the design of subsequent clinical trials.

#### KRAS

The rat sarcoma (RAS) proto-oncogene family frequently mutates in human tumors, with KRAS mutations being the predominant type, present in 90% of pancreatic cancer, 50% of colorectal cancer (CRC), and 30% of lung adenocarcinoma (LUAD)^[52]^. KRAS mutations constitute approximately 15% of all cancer patients^[53]^. The mutation rates in the University of Texas MD Anderson Cancer Center (MDACC) primary cohort were as follows: TP53 (60%), KRAS (37%), AR (21%), and STK11 (19%)^[54]^. KRAS is an oncogene of the MAPK signaling pathway, accounting for approximately 15%-30% of lung cancers^[55]^. Among them, LUAD and lung squamous cell carcinoma (LSCC) account for approximately 30% and 5%, respectively. PD-L1 expression is present in approximately 24%-55% of KRAS-mutated LUAD.

Chemotherapy is the usual course of treatment for individuals with KRAS mutations, resulting in an average OS of less than 2 years^[55]^. KRAS-mutated lung cancers are frequently distinguished by a significant quantity of T cell infiltrates that enhance neutrophil recruitment, leading to immune exclusion and T cell suppression^[56]^.

Liu *et al.* carried out a cohort study involving 74 patients with KRAS mutations who received ICIs and discovered that patients harboring the KRAS-G12D mutation exhibited a significantly shorter PFS compared to those with other mutations. Additionally, they found that KRAS-G12D mutant mice had a lower TMB, and PD-L1 levels were suppressed via the P70S6K/PI3K/AKT axis. Moreover, the combination of PD-L1 inhibitors and paclitaxel markedly enhanced the recruitment of CD8^+^ tumor-infiltrating lymphocytes (TILs), thereby inhibiting the tumor growth of lung adenocarcinoma in mice^[57]^. Concurrently, the KRAS-G12C mutation represents the most prevalent mutation site in NSCLC^[58]^. At present, several KRAS-G12C inhibitors, such as AMG510 and MRTX849, have received FDA approval, and others are in the clinical research stage. Mugarza *et al.* contend that KRAS-G12C inhibition leads to the upregulation of myc and the subsequent activation of the IFN signal, resulting in a decrease in the infiltration of immunosuppressive cells into the tumor, an enhancement of the infiltration and activation of cytotoxic T cells (CTLs), and an increase in antigen presentation. Nevertheless, KRAS-G12C inhibition fails to render “cold tumors” more sensitive to immunotherapy. Due to its effects on tumor immunity, it offers the potential for combining KRAS-G12C inhibitors with ICIs in subsequent treatments^[59]^.

Resistance to KRAS inhibitors has already emerged, and Zhang *et al.* identified that the majority of resistant patients retained the KRAS-G12C allele^[60]^. Hence, KRAS-G12C mutant cells treated with the covalent inhibitor ARS1620, which forms semi-antigenic MHC-I complexes, can be utilized as tumor-specific new antigens to evoke CTL responses against KRAS-G12C cells, thereby enhancing therapeutic efficacy.

#### Deletion of chromosome 9p

9p21.3 homozygous deletion, also known as 9p21 deletion, is a prevalent genetic abnormality found in around 13% of cancer cases. Han *et al.* undertook an analysis of genomic and transcriptomic data from 33 TCGA projects. They discerned that CDKN2A and MTAP were frequently absent or co-absent in the genes located on 9p21.3, accounting for approximately 9.2% of total deletions. Subsequent analyses of 9p21 deletions disclosed that they were correlated with a shorter OS. Additionally, it was elucidated that 9p21 deletions were associated with a lower clinical benefit. The immune phenotype was related to cold tumors, characterized by reduced tumor immune cell infiltration and a scarcity of innate immune cell infiltration. Consequently, it was determined that 9p21 deletion was associated with primary resistance to ICIs^[61]^. Therefore, non-squamous NSCLC with chromosomal 9p deletion has a unique tumor immune microenvironment and poor prognosis in response to ICIs^[62]^.

### Circular RNA axis and transcription factor

The circular RNA circ-CPA4 modulates the proliferation, motility, desiccation, and drug resistance of NSCLC cells through the let-7 miRNA/PD-L1 axis while also impairing CD8^+^ T cell function within the TME^[63]^. Overexpression of circ-CPA4 enhances PD-L1 expression, leading to PD-L1-mediated apoptosis and subsequent CD8^+^ T cell inactivation, thereby facilitating immune evasion. Additionally, numerous studies have demonstrated that elevated levels of the transcription factor YY1 are implicated in regulating tumor cell resistance to cellular immunotherapy^[64]^. Dillen *et al.* computed the pathway activity scores for 10 cancer-related pathways across 32 cancers and discovered that YY1 was demonstrated to potentially activate the cell cycle pathway in 31% of tumors. YY1 is associated with resistance to ICIs, and it can disrupt the PD-1/PD-L1 axis by activating the transcription [signal transducer and activator of transcription (STAT)], Janus kinase (JAK), phosphatidylinositide 3-kinases/protein kinase B (PI3K/Akt), and nuclear factor kappa-B (NF-κB) transcriptional pathways to undermine immunotherapy^[65]^.

### Antigen presentation disorder

Activating transcription factor-3 (ATF3), which downregulates cholesterol 25-hydroxylase (CH25H), is activated by stimuli generated from the TME. The advancement of lung cancer and tumor growth are linked to the absence of CH25H in antigen-presenting cells. Lu *et al.* measured the level of ATF3 in human monocytes and the level of ATF3 mRNA in mice and found that mice lacking CH25H in DCs had rapid tumor growth while CD8^+^ T cell activation in tumors was impaired. Therefore, inactivating ATF3 can increase CH25H levels, activate CD8^+^ T cells, and inhibit tumor growth. However, this pathway needs further discussion because ATF3 sometimes exhibits tumor-suppressive properties^[66]^.

### Signal pathway disorders

Trono *et al.* assessed the effects of hMENA^11a^ downregulation by means of RNA-sequencing, ATAC-sequencing, and flow cytometry and discovered that the deletion of hMENA^11a^ induced IFN-β production similar to that in aseptic inflammation. hMENA^11a^-mediated IFN-β signaling and secretion maintains an immunosuppressive TME, and thus, patients with NSCLC have a poorer prognosis when hMENA is expressed in cancer cells with low levels of epithelial-specific hMENA^11a^ isoforms. hMENA^11a^ downregulation mediates a possible novel mechanism of resistance to ICIs^[67]^.

Additionally, initial resistance to anti-PD-1/anti-CTLA-4 monoclonal antibody therapy is linked to the activation of the Wnt/β-catenin signaling cascade in several tumors^[68]^. Reduced expression of the C-C motif chemokine ligand 4 (*CCL4*) gene and loss of CD8^+^ T cells are linked to its activation. One of the most distinctive factors that drives cancer is Wnt/β-catenin signaling. By controlling the tumor immune cycle in the majority of lymph nodes, it accelerates the development of cancer. In particular, many regulators essential for T cell antitumor activity - effector T cells, helper T cells, and regulatory T (T_reg_) cells - are directly altered by aberrant Wnt/β-catenin signaling. By addressing Wnt/β-catenin signaling, immunotherapy resistance in cancer patients can be overcome, leading to better clinical outcomes^[69]^.

## ROLE OF THE TME IN NSCLC

**Figure 1:**
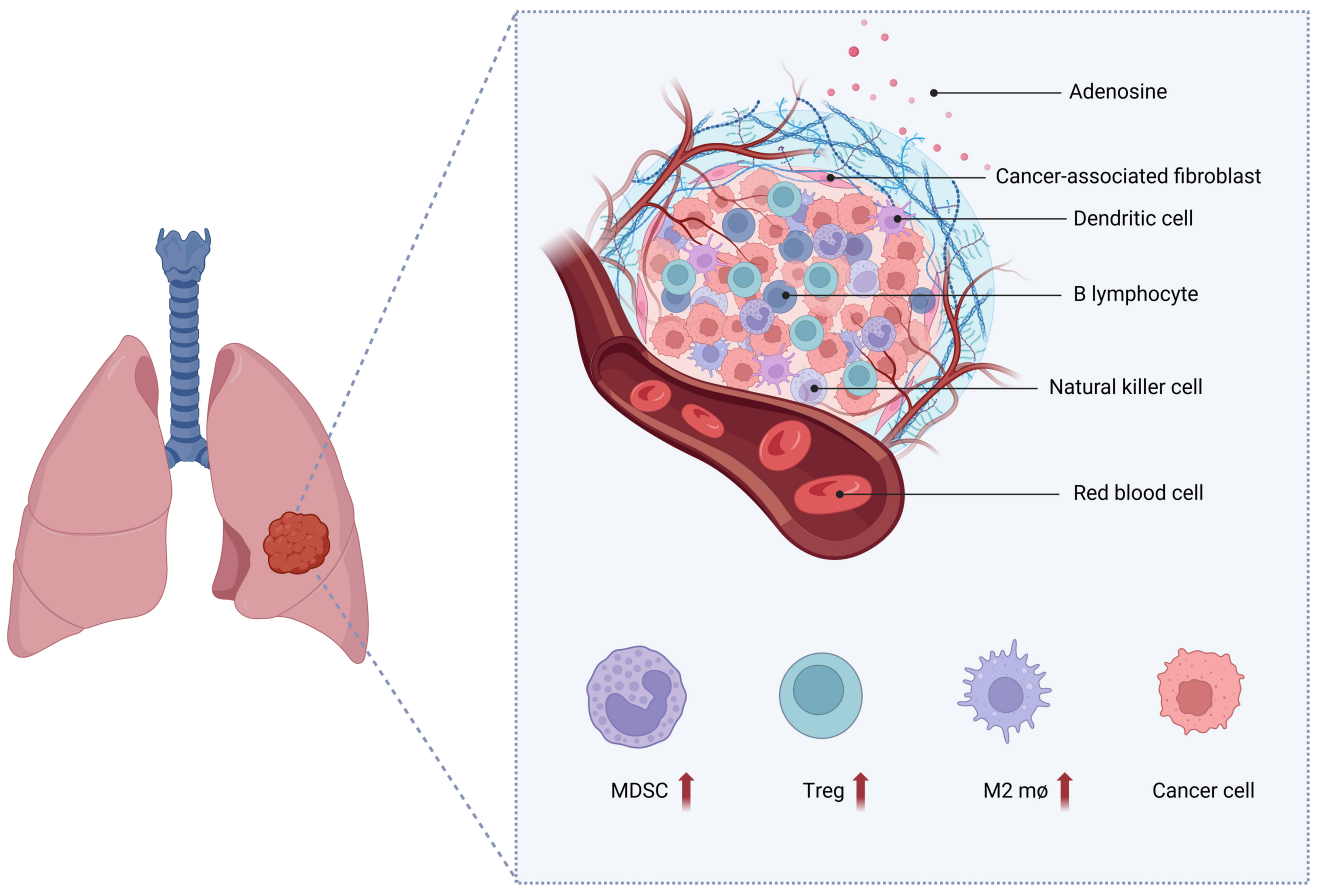
TAMs or MDSCs are considered to be important promoters of the TME. These cells can secrete cytokines, such as TGF-β, which activate primary-derived inflammatory pathways in the TME. Additionally, T_reg_ cells and extracellular adenosine can promote immunosuppression. Figure was created with Biorender.com. TAMs: Tumor-associated macrophages; MDSCs: myeloid-derived suppressor cells; TME: tumor microenvironment; TGF-β: transforming growth factor-beta; T_reg_: regulatory T; M2 mø: M2 macrophages.

TME is the internal milieu where tumor cells originate and exist. It comprises diverse cell types, such as immune cells and fibroblasts^[70]^. Immune cells within the TME engage in interactions with tumor cells, which have an impact on the advancement of malignancy, the ability of the tumor to evade the immune system, and its resistance to therapy^[71]^. Neutrophils, T cells, TAMs, and myeloid-derived suppressor cells (MDSCs) are recognized as significant contributors to the TME. These cells have the ability to release cytokines, including transforming growth factor-beta (TGF-β), that trigger primary-derived inflammatory pathways in the TME^[72]^ [Figure 1].

In addition, extracellular adenosine, which is usually present in the TME, promotes immunosuppression mainly through adenosine 2a receptor (A2aR) expressed by immune cells^[73,74]^. The TME facilitates the evasion of tumor cells from the cytotoxic effects of conventional antitumor therapy by orchestrating several processes, ultimately leading to the development of drug resistance^[75]^. Specific pathways or genes in tumor cells can prevent the infiltration of immune cells or inhibit the immune microenvironment, making it difficult for immunotherapy to play its role, leading to the occurrence of immune drug resistance. These factors can lead to the initiation of driving genes and the deactivation of tumor suppressor genes, the decrease of tumor antigen presentation ability, and the change in PD-L1 expression on the surface of tumor cells. We discussed the different manifestations of the TME according to primary drug resistance, adaptive drug resistance, and acquired drug resistance.

### Primary resistance

Primary resistance is mainly associated with inactivated tumor immunity and gene mutations, which can ascertain the immunotherapy’s responsiveness or refractoriness. The key mutations currently known are EGFR, KRAS, Serine-threonine kinase 11 (SKT11/LKB1), *etc.*, and co-mutations of these genes^[76]^.

#### EGFR

EGFR, as a driver gene in NSCLC patients, has been extensively studied. The TME of EGFR-mutant LUAD is commonly defined by an abundance of M2-polarized macrophages, reduced levels of CD8^+^ cells, elevated numbers of T_reg_ cells, and upregulated PD-L1 expression, all contributing to immune evasion^[77]^. In NSCLC cells with EGFR mutations, activated EGFR can upregulate PD-L1 expression through either the IL-6/JAK/STAT3 signaling pathway or the p-ERK1/2/p-c-Jun pathway^[78,79]^. Furthermore, EGFR mutations have been related to immune tolerance and weak immunogenic non-inflammatory TME, leading to poor response to PD-1 blockade^[80]^. Notably, CheckMate 078^[33]^, KEYNOTE-021^[26]^, KEYNOTE-024^[22]^, and KEYNOTE-042^[24]^ excluded NSCLC patients with EGFR/ALK sensitive mutations. Therefore, additional research is required to examine the correlation between EGFR mutations and the effectiveness of immunotherapy.

Activation of EGFR-related pathways can regulate immune cells and cytokines in the TME and confer immunotherapy resistance. In NSCLC tissues, NSCLC cells induce immunoglobulin-like transcript 4 (ILT4) expression by activating EGFR-AKT and ERK1/2 signaling cascades. Elevated levels of ILT4 hinder tumor immunity. Conversely, inhibition of ILT4 enhances the efficacy of PD-L1 inhibitors in EGFR WT NSCLC instead of EGFR mutant NSCLC^[81]^. Furthermore, the PI3K/AKT pathway mediated by EGFR activation is implicated in metabolic reprogramming, converting effector CD8^+^ T cells into memory T cells^[82,83]^.

#### KRAS/STK11

Co-mutations of KRAS/STK11 account for 25% of KRAS-mutated NSCLC and are characterized by lower levels of PD-L1. STK11 mutations are the main genomic driver causing primary resistance to ICIs^[84]^. Therefore, alterations in the *STK11* gene are considered poor predictors for response to immunotherapy. Ricciuti *et al.* conducted genomic data analysis on 1,261 patients with advanced lung adenocarcinoma and identified that 20.6% and 19.2% of the patients presented with detrimental mutations in STK11 and KEAP1, respectively. Mutations in STK11 and kelch-like ECH-associated protein 1 (KEAP1) were associated with conspicuously lower PD-L1 expression in LUAD patients and were correlated with inferior ORR, mPFS, and mOS following ICI treatment. Hence, the *STK11* gene mutation is intimately related to suboptimal ICI treatment outcomes. Simultaneously, LUAD patients with STK11 and KEAP1 mutations also demonstrated poorer responses to platinum-based chemotherapy^[85]^.

Additionally, mutations in LKB1 (STK11) play an important role in the sensitivity of tumors to ICB. Bai *et al.*’s study utilizing single-cell RNA sequencing data revealed that cancer cells with LKB1 mutations significantly suppress the expression of intercellular adhesion molecule-1 (ICAM1). However, reintroducing ICAM1 into LKB1-deficient tumors reactivates interactions between tumor cells and effector cells, leading to enhanced homing and activation of CD8^+^ T cells and resensitization of the tumor to ICB. Therefore, ICAM1 expressed on tumor cells plays a pivotal role in coordinating antitumor immune responses, particularly adaptive immune responses^[86]^.

#### T cells

TME encompasses TILs, including CD4^+^ helper cells, immunosuppressive T_reg_ cells, and CD8^+^ CTLs. The distribution of systemic CD8^+^ T cells could be a potential biomarker for ICI therapy, but its distribution is highly heterogeneous and lacks specificity^[87]^. Primary or secondary drug resistance is usually associated with a high number of T_reg_ cells. T_reg_ cells are a specific group of T cells that have the ability to suppress the immune response to tumors, hence facilitating the growth and advancement of the tumor. T_reg_ cells, as a key factor in resistance to ICIs, are mainly found in the context of immune exclusion or immune-enriched fibrotic conditions^[88]^. Indoleamine-2,3-dioxygenase (IDO) is an important promoter of the proliferation and activation of T_reg_ cells and MDSCs^[89]^. IDO1 is expressed in all tissues or cells, with predominant localization in the intestine, lung, and reproductive tract. Macrophages and DCs express it, and its transcription is primarily regulated by IFN-γ^[90]^. The quality of T_reg_ cells is related to the adverse reactions of ICB. Specifically, IFN-γ-dependent Th1-like T_reg_ cells are closely related to adverse reactions, while the number of T_reg_ cells, or the ratio of CD8^+^/T_reg_ cells, is not related to the adverse reactions of ICB^[91]^. T_reg_ cells exert immunosuppressive effects in cancer, and it is mediated by cell-intrinsic PD-1. The clinical application of anti-PD-1/PD-L1 disrupts this type of control, resulting in increased immunosuppression and the possibility of drug resistance in T_reg_ cells^[92]^.

Fortunately, CD39 is abundantly expressed in CD8^+^ T cells, and it can be a potential alternative to tumor-reactive CD8^+^ T cells^[93]^. ICB increases the number of tumor CD39^+^CD8^+^ T cells, and the genetic profile of CD39^+^CD8^+^ T cells can predict the efficacy of ICB^[93]^. Similarly, the OAK trial^[38]^ demonstrated that CD39^+^CD8^+^ T cell genetic profiles also predicted the outcome of ICB therapy.

In contrast, interleukin-6 (IL-6) production activates JAK1, which enlists and triggers the signal transducer and activator of the STAT3 pathway. The JAK1/STAT3 cascade is a key regulator of immune response and tumorigenesis, and IL-6 induces immune evasion by enhancing PD-L1 expression in tumors in this way. In addition, feedback activation of STAT3 triggered by secretion of IL-6 is also correlated with targeted drug resistance^[94]^. IL-6 levels have a negative correlation with CD8^+^ T cells and a positive correlation with myeloid-derived suppressor cells, M2 macrophages, and T_reg_ cells. Hence, the use of specific IL-6 inhibitors could potentially improve the effectiveness of anti-PD-L1 treatment in NSCLC^[95]^. In addition, C-reactive protein (CRP) is a readily available prognostic biomarker in ICIs for NSCLC. Elevated plasma A2aR levels are closely correlated with high baseline levels of plasma CRP and IL-6, which may have an impact on prognosis^[96]^.

In solid cancers, systemic inflammation is a relevant condition that influences tumor response in ICI therapy^[97]^. Thus, in inflammatory tumors with elevated IL-6 and TNF-α, myelopoiesis is more affected^[98]^. This may lead to altered cell counts in the blood, where neutrophils and other immune cells also secrete pro-inflammatory cytokines. Assessment of immune cell counts in serum can, therefore, be used to predict ICI efficacy^[99]^.

#### M2-TAMs

The accumulation of TAMs in the TME of NSCLC is related to resistance to immunotherapy. This phenomenon is caused by the increased expression of the CD27, integrin subunit alpha M (ITGAM), and *CCL5* genes within tumor compartments. Integrin (ITGAM or CD11b), chemokine (CCL5), and receptor (CD27) encode proteins, which may serve as potential targets for immunotherapy^[100]^. TAMs are important in remodeling the TME and promoting tumor growth, immunosuppression, and angiogenesis. TAMs function similarly to M2 macrophages by inhibiting the activity of CD8^+^ T cells and causing NK T cell dysfunction through the expansion of T_reg_ cells. This expansion of T_reg_ cells indirectly suppresses effector T cells, thereby reducing the number of antitumor immune cells and accelerating tumorigenesis^[101-103]^. The expression of xCT (SLC7A11) is notably increased in TAMs of lung cancer, demonstrating that xCT may have a function in regulating the TME of lung cancer. Higher xCT levels in TAMs were correlated with an unfavorable prognosis and served as a standalone predictor of lung cancer. Macrophage xCT deletion inhibits the activation of AKT/STAT6 signaling, which reduces M2-type polarization in TAMs. Thus, greater tumor suppression was obtained when anti-PD-L1 and macrophage xCT deletion were combined. In brief, inhibition of xCT has a greater potential in overcoming resistance to tumor immunotherapy^[104]^.

#### VEGF

Vascular endothelial growth factor (VEGF) drives the immunosuppressive action of effector T cells. It also induces vascular abnormalities, inhibits antigen presentation, and enhances T_reg_ cells, MDSCs, and TAMs^[105]^. Recruitment of MDSCs to tumors can be triggered by several stimuli, including colony-stimulating factor 3 (CSF-3), IL-1b, and IL-6. This recruitment process then activates STAT3, causing MDSCs to become cells that promote angiogenesis and inhibit the immune system^[106]^. Furthermore, TME immune cells can stimulate tumor angiogenesis. Conversely, immunosuppressive cells can stimulate angiogenesis and the formation of new blood vessels. This can lead to a harmful loop where antitumor immune responses are suppressed. In the thymus, VEGF prevents hematopoietic stem cells from differentiating into CD4^+^ and CD8^+^ T lymphocytes^[106]^. VEGF inhibits T cell proliferation and cytotoxicity by binding to VEGF R2 on T cells, which upregulates PD-1, PD-L1, and CTLA-4, thereby inhibiting T cell activation^[107]^.

Ang-2 is another important immunomodulator of vascular growth. It hinders the release of TNF-α, thereby restricting monocytes’ cancer-fighting capabilities^[108]^. All things considered, the immunosuppressive TME can result from anomalies in the tumor vasculature.

#### Pulmonary fibrosis

Massive fibrosis in the lungs of NSCLC patients is connected with reduced T cell infiltration, and the extent of interstitial fibrosis has an inverse relationship with T cell responses and results in immunosuppression within the TME. These changes are partly connected with TGF-β signaling. Lung fibrosis results in a fast progression of lung cancer, compromised T cell immune monitoring, and ineffectiveness of ICB treatments. Therefore, antifibrotic therapy may be a candidate strategy to overcome immunotherapy resistance. The finding showed that by specifically addressing fibrosis through TGF-β receptor signaling, it was possible to counteract the negative consequences of fibrosis, elevate T cell responses, and increase the effectiveness of ICB. However, this result was only effective in combination with chemotherapy, which indicated that it might be a promising strategy. The benefits of this approach are directly related to fibrosis^[70]^.

#### MAIT cells

Shi *et al.* collected serum samples from NSCLC patients both before and after anti-PD-1 therapy to investigate the distribution, function, and correlation with anti-PD-1 immunotherapy of MAIT cells^[109]^. They found no difference in the frequency of MAIT cells before and after anti-PD-1 treatment, nor between patients with cancer and healthy individuals^[110]^, and the function of MAIT cells was closely related to ICB therapy. Tumor-infiltrating MAIT cells presented an exhausted phenotype featuring upregulated PD-1 and IL-17A and downregulated IFN-γ. Exhausted MAIT cells produced high levels of IL-17A, which was associated with a poor prognosis. Furthermore, IL-17A could increase PD-L1 expression and facilitate tumor immune evasion. Simultaneously, tumors with a high content of MAIT cells were associated with an unfavorable prognosis. Additionally, two studies^[111,112]^ demonstrated that a high infiltration of MAIT cells in hepatocellular carcinoma (HCC) was significantly correlated with poor clinical outcomes and that serum CEA levels in CRC patients were positively correlated with tumor-infiltrating MAIT cells but negatively correlated with circulating MAIT cells in advanced CRC patients, and that the percentage of tumor-infiltrating MAIT cells in advanced CRC patients increased. The administration of anti-PD-1 therapy reversed the activity of circulating MAIT cells, leading to an increase in the production of IFN-γ. In NSCLC patients responding to anti-PD-1 therapy, the circ-MAIT-IFNGR cell population expanded, restoring effector function and cytotoxic ability. The circulating MAIT subpopulation might be a predictor of a favorable response to anti-PD-1 immunotherapy and is associated with primary resistance^[109]^. An initial study with a sample size of 70 patients^[113]^ calculated the percentage, partial function, and clinical relevance of circulating MAIT cells in lung cancer patients, and found that the concentration of activated CD38^+^CD8^+^MAIT cells in the peripheral blood of lung cancer patients was elevated, and it was positively correlated with IL-6, indicating an immunosuppressive function and affecting the PFS of patients.

### Adaptive resistance

Adaptive resistance occurs in the TME, where tumor cells undergo evolutionary changes in response to therapeutic interventions, leading to a reduction in new antigen production and mutational burden. They adapt to immune attacks, evading antitumor responses. This adaptation diminishes the tumor's sensitivity to adaptive immune reactions and induces inherent alterations within tumors, such as modifications in the expression of immunomodulatory molecules on tumor cells^[114]^. Clinically, adaptive resistance can manifest as primary resistance, mixed response, or acquired resistance^[17]^. Currently, there is limited research on the specific mechanisms underlying adaptive resistance. Therefore, detailed explanations will not be provided here. Related content has been introduced in primary resistance and acquired resistance.

### Acquired resistance

Acquired resistance is observed in solid tumors with high tumor burden and immune cell infiltration that initially respond well to therapy. However, the continuous evolution of tumor cells enables them to evade recognition by the immune system and mediate immunosuppression. Additionally, long-term secretion of soluble molecules by tumor cells recruits inhibitory immune cells to the site of the tumor. Upon interaction with these cells, a compensatory inhibition mechanism is established, which promotes tumor growth and metastasis while facilitating angiogenesis and immune evasion. Consequently, some patients who initially respond positively to immunotherapy eventually develop acquired resistance that impacts their overall treatment outcome^[114]^. The mechanisms behind acquired resistance are currently under investigation.

#### “Cold” and “hot” tumors

Research related to “cold” and “hot” tumors has been a hot topic. “Cold” tumors are characterized by minimal infiltration of CTLs and high levels of MDSCs and TAMs. Therefore, “cold” tumors are highly resistant to conventional immunotherapies, including ICIs. The immune characteristics of the TME are important factors affecting the efficacy of tumor immunotherapy. “Cold” tumors, which are enriched with immunosuppressive cell populations, are particularly resistant and require urgent intervention to overcome this resistance to treatment^[115]^. In contrast, a recent study has demonstrated that “hot” tumors driven by IFN-γ are susceptible to acquired resistance against PD-1 blockade. Instead of exhibiting immune rejection or desert phenotypes, most resistant “hot” tumors maintain an inflammatory profile. During this stage, both IFN-1 and IFN-2 are significantly upregulated, accompanied by an increased number of CD8^+^ T cells. Consequently, the propensity for acquired resistance in “hot” tumors is closely linked to the activation of the IFN-γ signaling pathway. This finding holds great significance for informing future immunotherapy strategies. Nonetheless, its generalizability requires further investigation^[116]^.

#### IFN-γ pathway

IFN-γ exhibits pleiotropic effects, including antiproliferative, proapoptotic, and antitumor mechanisms. However, recent evidence suggests that it may also promote tumor growth through insensitivity to IFN-γ signaling, downregulation of the MHC, and upregulation of IDO and checkpoint inhibitors^[117]^. Our current understanding of acquired resistance to ICIs remains limited. Acquired resistance is characterized by altered expression of IFN signaling pathways. Recurrent tumors can be distinguished by increased or stable expression of genes responsive to IFN-γ. Upregulation of these genes is associated with well-established resistance mechanisms such as sustained IFN signaling, immune dysfunction, and mutations in antigen-presenting genes^[116]^.

#### EVs

EVs are currently recognized as one of the primary modes of intercellular communication within the TME, playing a pivotal role in regulating tumor resistance. EVs possess the capacity to facilitate communication between cancer cells and the TME, thereby influencing tumor transformation, progression, and metastasis^[118]^. They include microvesicles (MVs), apoptotic vesicles (apoVs), and exosomes^[119,120]^. For example, TGF-β is available in the circulation and is packaged into EVs with specific delivery properties that can influence the interaction between the immune system and cancer. It is an immunosuppressive cytokine, and studies have demonstrated that higher baseline levels of TGF-β in EVs correlate with poorer responses to ICIs and shorter PFS/OS, making TGF-β a better predictive biomarker^[121]^.

#### Oxidative stress

Oxidative stress is caused by high levels of reactive oxygen species (ROS) due to intense metabolism and oxygen consumption. ROS is produced by mitochondria and is the main mediator of tumor proliferation, metastasis, and immune escape. Therefore, additional TME components may potentially be impacted by the dynamic equilibrium of oxidative stress. For instance, changes to T cells, DCs, and M2 macrophages result in immunosuppression. Mitochondria generate mitochondrial reactive oxygen species (mtROS), which triggers the IFN signal, upregulates PD-L1 expression, and inhibits T cell activation. Meanwhile, high levels of ROS promote T cell apoptosis and have an impact on the antigen-presenting capacity of DC cells. Concurrently, under the stress of ROS, cancer cells secrete IL-6, IL-4, and other inflammatory cytokines to facilitate immune suppression, increasing the likelihood of TAMs differentiating into the M2 type^[122,123]^.

#### Gene mutation after treatment

It has been shown that STK11 mutation may occur in patients with advanced LSCC and Lynch syndrome following the administration of pembrolizumab as initial treatment and achieving SD, leading to PD. This patient’s plasma circulating tumor DNA (ctDNA) revealed the presence of a novel STK11 frameshift mutation. Therefore, STK11 may lead to acquired resistance to pembrolizumab^[124]^.

#### CRD

The human immune function is modulated by the circadian rhythm, and the concentration of immune-related cytokines undergoes significant fluctuations throughout the day. Moreover, the circadian rhythm constitutes the core of the body’s immune response against pathogens^[125]^. Studies have demonstrated that CRD is associated with drug resistance. CRD makes a substantial contribution to T cell exhaustion, and the expression of the key cell cycle protein D1 (CCND1) is conspicuously augmented in highly malignant cells within CRD. It is intimately related to poor prognosis and drug resistance in LUAD patients. Roberts *et al.* discovered that the growth rate of mouse tumors was more rapid than that under standard lighting by manipulating external light sources to disrupt the mouse’s circadian rhythm^[126]^. He *et al.* developed an algorithm capable of calculating the CRD score to predict patients’ responses to ICI treatment. Currently, the pharmacological control employing circadian rhythm genes can serve as an innovative therapeutic approach for cancer combination therapy^[127]^.

Gettinger *et al.* researched ICI-resistant lung cancer samples and identified a homozygous B2M deletion in tumors resistant to PD-L1 and anti-CTLA-4 therapy. B2M is a crucial element of the human leukocyte antigen-I (HLA-I) complex, and tumor B2M deletion is associated with immune evasion, which is one of the adverse prognostic factors in various cancers^[128]^. B2M deletion is considered to be irreversible, and it is the primary resistance mechanism. These findings indicate that disruption of the HLA-I antigen processing and presentation mechanism (APM) can mediate ICI escape in lung cancer. For instance, activated immune cells within an inflammatory milieu can secrete IFN-γ, which serves as a crucial pathway for regulating HLA-I and human leukocyte antigen-II (HLA-II) presentation. Disrupting the signaling of IFN-γ will give rise to immune evasion^[129]^. The administration of entinostat plus NHS-IL12 treatment leads to the stimulation of M1-like macrophages and activated antigen-presenting cells while reducing the presence of M2-like macrophages and T_reg_ cells. Additionally, this treatment stimulates the upregulation of MHC-I and APM, which in turn triggers a strong antitumor response and improves survival rates^[130]^.

Paulson *et al.* discovered that drug-resistant tumors exhibit dynamic transcriptional inhibition of specific *HLA* genes targeting viral epitopes, which is attributed to robust CD8-mediated immune pressure. This phenomenon differs from hereditary HLA deletion as it can be reversed with the use of drugs. Transcriptional repression at class I loci may be a potential mechanism for immunotherapy resistance^[131]^.

Hypoxia constitutes a characteristic hallmark of the TME. Hypoxia exerts a substantial influence on tumor progression and metastasis, which is triggered by the uncontrolled proliferation of tumor cells and the development of abnormal vascular formation^[132,133]^. In hypoxic environments, physical barriers conducive to tumor survival are established by regulating the evasion of tumor cells from the immune response^[134]^. The activation of genes associated with low oxygen levels, such as VEGF, hypoxia-inducible factor-1α (HIF-1α), glucose transporters (GLUTs), and basic fibroblast growth factor, is augmented within the TME. This exacerbates the angiogenesis and cell growth processes, ultimately resulting in cancer progression and both primary and acquired resistance. As a byproduct of glycolysis, hypoxic cells are accompanied by elevated levels of intracellular lactic acid and hydrogen ions. Owing to high metabolic activity and insufficient perfusion, acidic metabolic waste accumulates within the TME. The acidity of the TME may impact tumor immune surveillance and ultimately give rise to immune evasion and cancer progression. The acidic environment can inhibit the antitumor immune functions of M1 macrophages, T cells, DC cells, and NK cells, and enhance the immune inhibitory functions of M2 macrophages and T_reg_ cells^[135]^. Preliminary evidence indicates that T cells and NK cells frequently lose functionality when exposed to an acidic environment, followed by cell apoptosis^[136]^. Conversely, immune inhibitory components bind to the acidity of the tumor to sustain tumor growth and block antitumor immune responses^[136]^.

**Figure 2:**
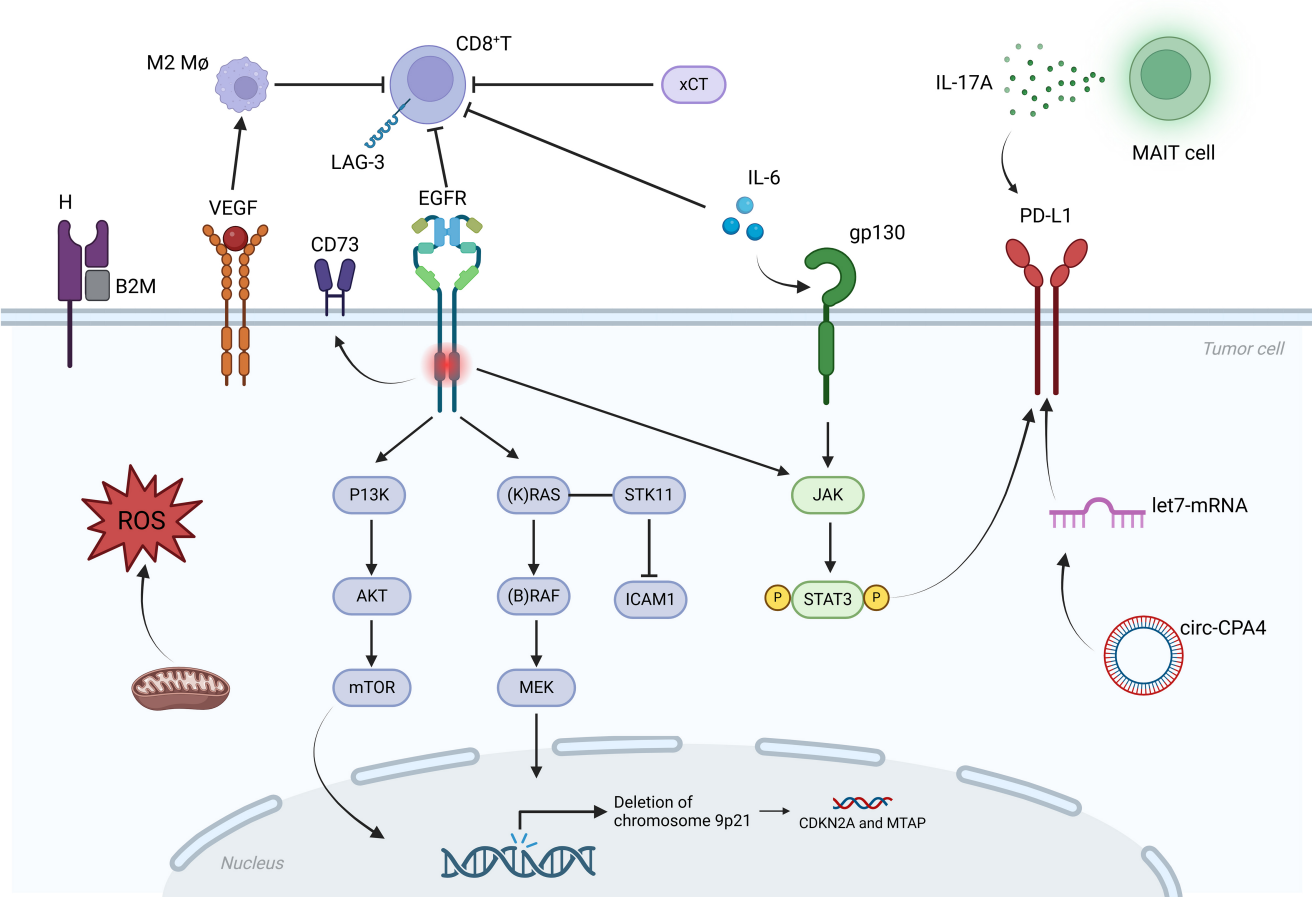
Mechanisms of NSCLC immunotherapy resistance. In primary resistance mechanism, KRAS/STK11 co-mutation is the main genomic driver of primary ICI resistance, which reduces lymphocyte infiltration and PD-L1 expression. EGFR is the upstream signaling pathway of KRAS. When EGFR is activated, ILT4 levels rise, which increases the recruitment of M2-TAMs and inhibits immune function. EGFR mutant tumor cells inhibit T cell proliferation and associated functions and exhibit a significant increase in resistance to T cell-mediated killing. In contrast to EGFR WT NSCLC, EGFR mutant tumor cells express a higher level of CD73, and the LAG-3 receptor expressed on T cells is notably upregulated following TKI treatment. At the same time, circ-CPA4 regulates the growth, migration, dryness, and drug resistance of NSCLC cells through the let-7 miRNA/PD-L1 axis. Exhausted MAIT cells produced high levels of IL-17A, which was associated with a poor prognosis. The loss of chromosome 9p21 is associated with primary resistance to ICI and CDKN2A, and MTAP deletions account for about 9.2%. IL-6 can activate the JAK1/STAT3 pathway to enhance PD-L1 expression. The level of IL-6 is negatively correlated with CD8^+^ T cells. VEGF can induce M2 macrophages to enhance the immunosuppressive effect. The overexpression of xCT in TAMs participates in the regulation of the TME, which can promote M2 polarization and inhibit immunity. Mitochondria produce ROS, promote oxidative stress, and immunosuppressive components combine with tumor acidity to maintain tumor growth and block antitumor immune response. In addition, the B2M deletion of HLA-I complex is also associated with immune escape. Figure was created with Biorender.com. NSCLC: Non-small cell lung cancer; KRAS: Kirsten rat sarcoma viral oncogene homolog; STK11: serine/threonine kinase 11; ICI: immune checkpoint inhibitor; PD-L1: programmed cell death-ligand 1; EGFR: epidermal growth factor receptor; ILT4: immunoglobulin-like transcript 4; M2-TAMs: M2 tumor-associated macrophages; WT: wild-type; LAG-3: lymphocyte activation gene-3; TKI: tyrosine kinase inhibitor; MAIT: mucosal-associated invariant T; MTAP: methylthioadenosine phosphorylase; IL-6: interleukin- 6; JAK1: Janus kinase 1; STAT3: signal transducer and activator of transcription 3; VEGF: vascular endothelial growth factor; TME: tumor microenvironment; ROS: reactive oxygen species; HLA-I: human leukocyte antigen-I; M2 Mø: M2 macrophages; P13K: phosphatidylinositol-3-kinase; AKT: protein kinase B; mTOR: mammalian target of rapamycin; ICAM1: intercellular cell adhesion molecule-1.

In addition, assessment of changes in exosomal cytokine expression levels can better predict the relevance of tumor response to ICI therapy. Exosomal PD-L1 was found to be significantly downregulated, while exosomal PD-1 and IFN-γ were significantly upregulated in patients responding to ICIs^[15]^. The relationship between various drug resistance mechanisms and alterations in the TME is illustrated in Figure 2.

## STRATEGIES FOR OVERCOMING IMMUNOTHERAPY RESISTANCE

### Immunotherapy combination

Immunotherapy in combination with other treatments constitutes the principal response strategy in clinical practice, encompassing dual immunotherapy, the combination of immunotherapy with chemotherapy, and the combination of immunotherapy with anti-angiogenesis. Checkmate 9LA was devised based on the scheme of Checkmate 227 and involved two cycles of chemotherapy, in conjunction with PD-L1 and CTLA-4 inhibitors, which could conspicuously prolong the OS of patients in comparison to single chemotherapy. In contrast to ICI monotherapy, the dual blockade of PD-1/PD-L1 and CTLA-4 inhibitors operates at diverse stages of T cell activation and synergistically augments the immune response against cancer cells^[137]^. Chemotherapy can inflict DNA molecular damage upon tumor cells, thereby suppressing tumor growth and even eliminating tumor cells. The combination of immunotherapy with chemotherapy can augment the formation of new antigenic complexes, promote the immunogenicity of cancer cells, induce immune cell death, modify the cytokine environment within the TME, and result in the redistribution and increased expression of PD-L1 on tumor cells^[138]^. The BTCRC-LUN15-029 trial demonstrated that in comparison with the previous data of single chemotherapy, chemotherapy combined with pembrolizumab for advanced NSCLC patients with PD after ICIs could yield PFS (6.9 mo) and OS (26.8 mo) benefits.

Pathological angiogenesis within tumors assumes a crucial role in tumor progression, giving rise to an immunosuppressive TME and accelerating tumor advancement. The aberrant distribution and dysfunction of tumor blood vessels result in hypoxia and acidity within the TME, rendering it challenging for immune cells to infiltrate the tumor area. These factors can significantly influence the response to immunotherapy^[139]^. Hence, vascular normalization can effectively enhance the TME and offer opportunities to overcome resistance to ICIs. Anti-angiogenic drugs exert immunomodulatory effects within the TME and can reverse the immunosuppression induced by tissue hypoxia and immunosuppressive cells, enhance the maturation of DCs, as well as the transport and function of T cells. Simultaneously, it transforms the TME from an immunosuppressive type to an immuno-supportive one^[138]^. In IMpower150, targeting NSQ patients^[140]^, the combination of anti-VEGF monoclonal antibody bevacizumab, atezolizumab, and chemotherapy as first-line treatment significantly prolonged the OS of patients (19.5 mo *vs.* 14.7 mo).

### Tumor cell vaccine

Recently, the development of immune vaccines and small-molecule drugs has made great achievements^[141]^. Noticeably, the lung cancer cell vaccine, modified with Newcastle disease virus (NDV), encourages both *in vivo* and *in vitro* T cell activation and DC cell maturation. Additionally, it boosts the development of Th1 cells and infiltration of inflammatory cells *in vivo*. Furthermore, the administration of a lung cancer cell vaccine that has been modified with NDV, along with the injection of NDV directly into the tumor, has been shown to effectively suppress tumor growth, promote the differentiation of Th1 cells, and increase the infiltration of inflammatory cells *in vivo*. It also effectively activates T cells and upregulates IFN-γ and IL-4 in T cells, which results in a good immunotherapeutic effect. In brief, the utilization of NDV-modified lung cancer cell vaccination in conjunction with NDV tumor injection has demonstrated a noteworthy impact, offering a hopeful approach for the combined treatment of tumor immunotherapy^[142]^.

Messenger RNA (mRNA) tumor vaccines have shown surprising results in the treatment of lung cancer and bone metastases. When administered intranasally, mPLA/mRNA tumor vaccine (mLPR) reorganizes macrophages through the TLR4/JAK2/STAT1 pathway and polarizes M2 macrophages into M1 macrophages^[143]^. mLPR activates immune cells and secretes IFN-γ/IL-12 cytokines to inhibit lung cancer and reduce bone metastasis so that the mLPR tumor vaccine will provide information and possible uses for mRNA tumor vaccines in the management of lung cancer^[144]^.

### Small molecule drug

Nowadays, vaccines are developing rapidly, while small-molecule drugs have been found to improve the efficacy of ICI therapy. PIK-93^[145]^ is a small-molecule drug that regulates the TME, which enhances the antitumor cytotoxicity of M1 macrophages. It can enhance T cell activation, efficacy of therapy, and inhibit the growth of tumors when used with anti-PD-L1.

Furthermore, the DNA damage response (DDR) integrates multiple cellular regulatory processes and assumes a crucial role in maintaining the stability and integrity of the genome^[146]^. Ataxia telangiectasia and Rad3-related (ATR) constitutes a key kinase within the DDR core, and the principal function of ATR is to regulate DNA replication and activate cell cycle checkpoints to guarantee the integrity and completeness of cell division^[147]^. Currently, ATR inhibitors such as ceralasertib are increasingly utilized in clinical trials. The findings of the Hudson trial^[148]^ demonstrated that the combination of durvalumab and ATR inhibitor ceralasertib confers superior benefits to advanced NSCLC patients who have progressed after receiving ICI therapy, with an ORR of 13.9% *vs.* 2.6%, mPFS of 5.8 months *vs.* 2.7 months, and mOS of 17.4 months *vs.* 9.4 months. The Hudson trial remains ongoing, and new anti-cancer drug combinations will be incorporated in the future. Based on the results of the Hudson trial, the phase III LATIFY (NCT05450692) trial is currently in progress, which aims to compare the efficacy and safety of durvalumab combined with ceralasertib against docetaxel chemotherapy in NSCLC patients who have progressed after receiving ICIs and platinum-based chemotherapy. The remaining small molecule trials are also delineated in Table 2.

**Table 2:** Clinical trials investigating new treatments to overcome immunotherapy resistance in NSCLC

**Intervention/treatment**	**Drug/treatment**	**Target**	**ClinicalTrials.gov identifier**	**Phase**	**Patients**	**Recruitment status**
ICIs	WTX212A	PD-1	NCT06106152	I	ICI-naive and pretreated	Not yet recruiting
	TQB2450	PD-L1	NCT06141226	II	ICI-naive and documented PD after ICI treatment	Recruiting
	Alirocumab and cemiplimab	PCSK9, PD-1	NCT05553834	II	Documented PD after ICI treatment	Recruiting
	Gotistobart (ONC-392/BNT316)	CTLA-4	NCT05671510	III	Documented PD after ICI treatment	Active, not recruiting
	MRx0518 and pembrolizumab	PD-1	NCT03637803	I/II	Documented PD after ICI treatment	Terminated
	Cobolimab and dostarlimab	TIM-3, PD-1	NCT04655976	II/III	Documented PD after ICI treatment	Active, not recruiting
Radiotherapy	SBRT	/	NCT05387044	II	ICI-naive and documented PD after ICI treatment	Recruiting
TME modulators	L-TIL	/	NCT05878028	II	ICI-naive	Recruiting
	Oncobax-AK^®^	/	NCT05865730	II	ICI-naive	Recruiting
	NIZ985	IL-15/IL-15Rα	NCT04261439	I	ICI-naive and pretreated	Terminated
	MK-7684A	TIGIT	NCT04725188	II	evaluated PD after ICI treatment	Active, not recruiting
	Bintrafusp alfa	TGF-β	NCT04396535	II	ICI pretreated	Terminated
	Ipatasertib	AKT	NCT04467801	II	Including ICI-naive	Recruiting
Targeting-drive gene	Cobimetinib	MEK	NCT03600701	II	ICI-naive and pretreated	Active, not recruiting
Small molecule drugs	Ceralasertib, oleclumab, durvalumab	ATR, CD73, PD-L1, *et al.*	NCT03334617	II	Documented PD after ICI treatment	Active, not recruiting
	Ceralasertib, durvalumab	ATR, PD-L1	NCT05450692	III	Documented PD after ICI treatment	Active, not recruiting
	AL2846, TQB2450	c-Met, PD-L1	NCT05922345	III	Documented PD after ICI treatment	Recruiting
	M1774, cemiplimab	ATR, PD-1	NCT05882734	I/II	Documented PD after ICI treatment	Active, not recruiting
	N-803	IL-15	NCT03228667	II	Documented PD after ICI treatment	Active, not recruiting
	INCB106385	A2aR, A2bR	NCT04580485	I	Documented PD after ICI treatment	Active, not recruiting
Combined therapy	Pembrolizumab, ramucirumab, docetaxel	PD-1, VEGF	NCT04340882	II	ICI-naive and pretreated	Recruiting

NSCLC: Non-small cell lung cancer; ICIs: immune checkpoint inhibitors; PD-1: programmed death-1; PD-L1: programmed cell death-ligand 1; PCSK: proprotein convertase subtilisin/kexin; CTLA-4: cytotoxic T lymphocyte-associated antigen-4; TIM-3: T cell immunoglobulin domain and mucin domain-3; SBRT: stereotactic body radiotherapy; TME: tumor microenvironment; TIL: tumor-infiltrating lymphocyte; IL-15: interleukin-15; TIGIT: T cell immunoreceptor with immunoglobulin and ITIM domain; TGF-β: transforming growth factor β; AKT: protein kinase B; MEK: mitogen-activated extracellular signal-regulated kinase; ATR: ataxia telangiectasia and rad3-related protein; CD73: Ecto-5’-Nucleotidase; c-Met: cellular-mesenchymal to epithelial transition factor; A2aR: adenosine 2a receptor, A2bR: adenosine 2b receptor; VEGF: vascular endothelial growth factor.

### Nanomedicine

Numerous nanodrugs have been shown to mitigate the physical characteristics of the TME, including acidity and hypoxia. Hemoglobin (Hb) has been utilized as an oxygen carrier to enhance local oxygen concentration in tumor tissues. Similarly, nanomedicine designed with lactic acid oxidase or alkaline substances has been standardized to counteract the acidic TME caused by lactic acid accumulation^[75]^. Numerous other nanomedicine, including Nanomedicine SGT-53 and AZD1080, have demonstrated their ability to overcome immunotherapy resistance by enhancing tumor immunogenicity and promoting the release of CD8^+^ T cells, thus overcoming lung cancer resistance to ICB^[149]^.

### Epigenetic modifier

Toyokawa *et al.* discovered a positive correlation between PD-L1 and EZH2 in their evaluation of 428 patients who underwent resection for LUAD^[150]^. Therefore, an emerging strategy involves combining epigenetic modifiers with ICB to remodel the TME and enhance antitumor immunity. The combination of EZH2 inhibitors and PD-1 blockers presents a promising approach for patients with advanced lung cancer.

### Endocrine therapy

In female patients, estrogen receptor (ERa) is a predictor of pembrolizumab response, and ERa transcriptionally upregulates the *CD274/PD-L1* gene, which in turn can be activated by 17-β-estradiol produced by aromatase autocrine secretion. Thus, when pembrolizumab is combined with the aromatase inhibitor letrozole, its efficacy can be significantly improved. Aromatase inhibitors can be used as specific immune adjuvants in NSCLC^[151]^.

### Other treatments

**Figure 3:**
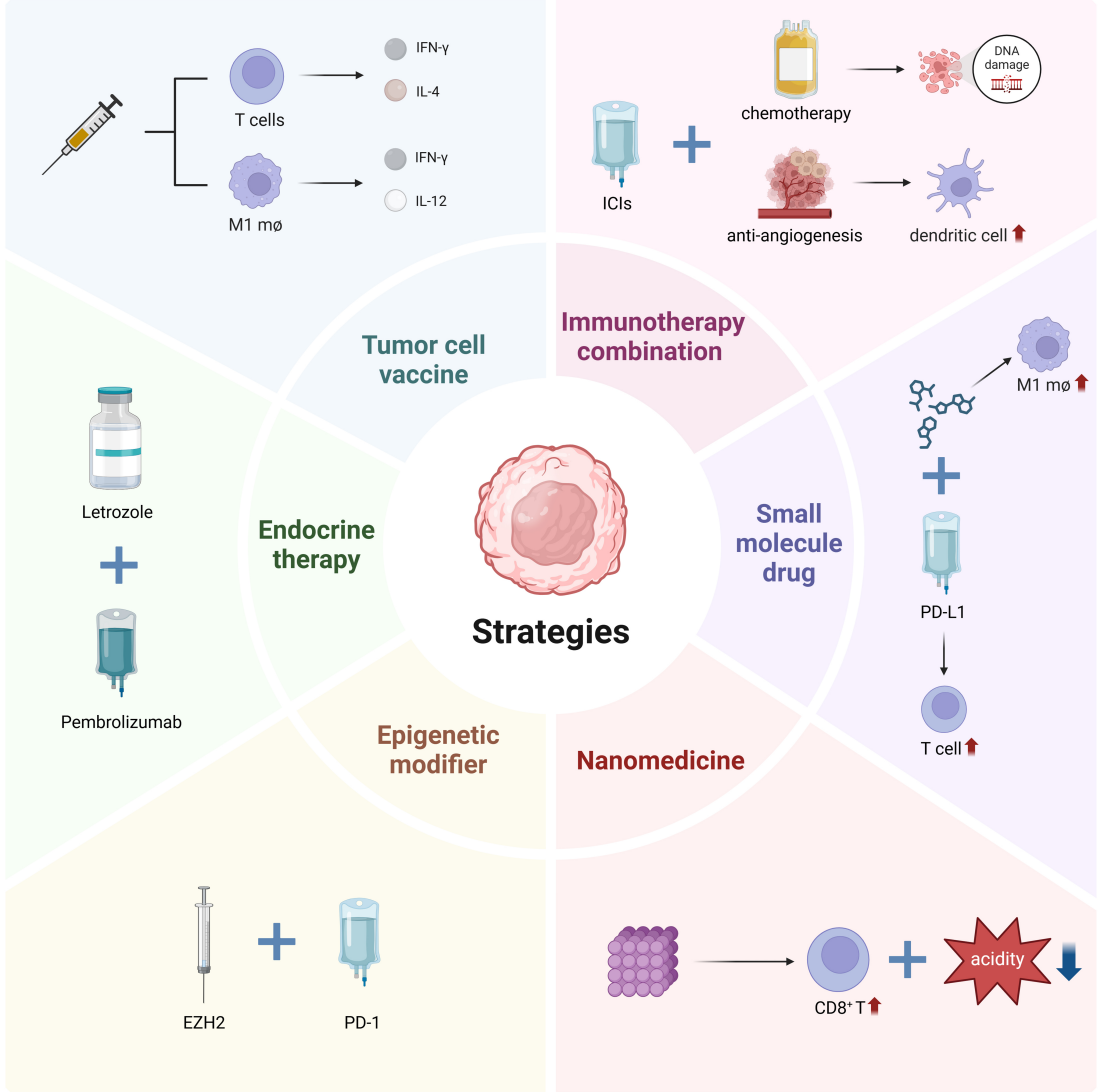
Mechanisms related to strategies for overcoming immunotherapy resistance. Immunotherapy, in combination with other treatments, constitutes the principal response strategy in clinical treatment. Chemotherapy can inflict DNA molecular damage upon tumor cells, thereby suppressing tumor growth and even eliminating tumor cells. The combination of immunotherapy with chemotherapy can augment the formation of new antigenic complexes, promote the immunogenicity of cancer cells, induce immune cell death, and modify the cytokine environment within the TME. Anti-angiogenic drugs exert immunomodulatory effects within the TME. They can reverse the immunosuppression caused by tissue hypoxia and immunosuppressive cells and enhance the maturation of DCs, as well as the transport and function of T cells. Tumor cell vaccines can effectively activate T cells and upregulate IFN-γ, IL-4, and IL-12, which result in good immunotherapeutic effect. Small-molecule drugs can enhance the antitumor cytotoxicity of M1 macrophages, and when combined with anti-PD-L1, they can enhance T cell activation, efficacy and inhibit tumor growth. Nanomedicine alleviates the physical properties of the TME and promotes the release of CD8^+^ T cells. Treatment efficacy can be enhanced when epigenetic modifiers are used with PD-1 inhibitors and aromatase inhibitors with pembrolizumab. Figure was created with Biorender.com. TME: Tumor microenvironment; DCs: dendritic cells; PD-L1: programmed cell death-ligand 1; PD-1: programmed death-1; ICIs: immune checkpoint inhibitors; EZH2: enhancer of zeste homolog 2.

The rest of the combination therapies, such as targeted therapy^[152]^, radiotherapy^[153]^, and traditional Chinese medicine^[154]^, have also shown better effects. Other factors such as M2 macrophages, T_reg_ cells, IDO^[155]^, T cell immunoreceptors with immunoglobulin, and ITIM domain (TIGIT)^[156]^ are all considered potential targets for other effective antitumor immune responses. We show several strategies for overcoming immunotherapy resistance in Figure 3. Relevant studies now underway to overcome immunotherapy resistance are shown in Table 2.

### Future prospects

The enrichment of TAMs in the TME of NSCLC is related to resistance to immunotherapy, potentially leading to the development of acquired resistance as TAM numbers increase. To overcome this resistance, cytokines can be utilized to modulate the phenotype of TAMs^[114]^. When a properly activated pool of repositionable T cells is present, a local equilibrium between effector T cells and tumors is achieved. However, at some point, this balance is disrupted, favoring tumor regrowth^[157]^. The mechanisms underlying immunotherapy resistance are still being extensively investigated, and gaining a fundamental understanding of the cellular and molecular processes involved in resistance is crucial for accurately targeting future combination therapies or those beyond anti-PD therapy^[158]^. Future studies on bio-reactive markers may focus on analyzing the composition of the entire TME rather than individual populations separately^[159]^.

## CONCLUSION

The development of immunotherapy has made gratifying achievements, and immunotherapy is now widely used in clinical treatment. However, primary and secondary resistance to immunotherapy reduces the efficacy of immunotherapy. The mechanism of immunotherapy resistance is not only related to the expression of biomarkers but also to the TME. The TME is influenced by multiple signaling pathways, leading to an upregulation of M2-TAMs, a downregulation of CD8^+^ T cells, and a rise in T_reg_ cells, which subsequently suppress the immune system. To overcome the resistance of immunotherapy, tumor cell vaccines, small-molecule drugs, nanomedicine, epigenetic modifiers, and endocrine therapy have demonstrated promising potential. Future research on biomarkers of reactivity will shift its focus from a single population to encompass the entire TME.

## DECLARATIONS

### Copyright

© The Author(s) 2024.
